# Germline mutation rate is elevated in young and old parents in *Caenorhabditis remanei*

**DOI:** 10.1093/evlett/qrad052

**Published:** 2023-10-17

**Authors:** Hwei-yen Chen, Therese Krieg, Brian Mautz, Cécile Jolly, Douglas Scofield, Alexei A Maklakov, Simone Immler

**Affiliations:** Department of Ecology and Genetics, Uppsala University, Uppsala, Sweden; Department of Biology, Lund University, Lund, Sweden; Department of Ecology and Genetics, Uppsala University, Uppsala, Sweden; Department of Medicine, Division of Epidemiology, Vanderbilt University, Nashville, United States; Department of Ecology and Genetics, Uppsala University, Uppsala, Sweden; Department of Organismal Biology, Uppsala University, Uppsala, Sweden; Department of Ecology and Genetics, Uppsala University, Uppsala, Sweden; School of Biological Sciences, University of East Anglia, Norwich Research Park, Norwich, United Kingdom; Department of Ecology and Genetics, Uppsala University, Uppsala, Sweden; School of Biological Sciences, University of East Anglia, Norwich Research Park, Norwich, United Kingdom

**Keywords:** mutation accumulation, age-related mutation rate, SNPs, parental age effects, germline mutations, selection shadow

## Abstract

The effect of parental age on germline mutation rate across generations is not fully understood. While some studies report a positive linear relationship of mutation rate with increasing age, others suggest that mutation rate varies with age but not in a linear fashion. We investigated the effect of parental age on germline mutations by generating replicated mutation accumulation lines in *Caenorhabditis remanei* at three parental ages (“Young T1” [Day 1], “Peak T2” [Day 2], and “Old T5” [Day 5] parents). We conducted whole-genome resequencing and variant calling to compare differences in mutation rates after three generations of mutation accumulation. We found that Peak T2 lines had an overall reduced mutation rate compared to Young T1 and Old T5 lines, but this pattern of the effect varied depending on the variant impact. Specifically, we found no high-impact variants in Peak T2 lines, and modifiers and up- and downstream gene variants were less frequent in these lines. These results suggest that animals at the peak of reproduction have better DNA maintenance and repair compared to young and old animals. We propose that *C. remanei* start to reproduce before they optimize their DNA maintenance and repair, trading the benefits of earlier onset of reproduction against offspring mutation load. The increase in offspring mutation load with age likely represents germline senescence.

## Introduction

Germline mutations are heritable DNA alterations in the germ cells that can be transmitted to future generations, and therefore are the ultimate source of genetic diversity and the fuel for evolution. The rate at which germline mutations arise is central to fields ranging from evolutionary biology to clinical research. The molecular clock, a key concept in genetics, is based on the assumption that the germline mutation rate, and hence the substitution rate, is constant (Zuckerkandl & Pauling, 1965). However, substitution rates vary substantially within and across species (Chintalapati & Moorjani, 2020; Moorjani et al., 2016) and depend on key factors such as sex (Hurst & Ellegren, 1998) and number of cell divisions (Lynch, 2010) as well as the relative age at maturation and age at reproduction (Amster & Sella, 2016; Crow, 2006; Ségurel et al., 2014). An evolutionary shift in the relative timing of these two events is likely to affect the estimate of germline mutation rate, and consequently substitution rate. A precise characterization of how germline mutation rate varies throughout life therefore has important implications in setting the evolutionary clock and in understanding the age-related inheritance of de novo mutations more generally (Moorjani et al., 2016; Scally, 2016). Empirical data estimating germline mutation rate as a function of parental age, however, are still scarce.

While it is known that parental age can affect germline mutation rate, exactly how germline mutation rate changes during an individual’s life is unclear. In mammals, the per cell division mutation rate during spermatogenesis is often assumed to be constant (Chang et al., 1994; Drost & Lee, 1995), and thus the number of germline mutations found in the offspring is expected to increase linearly with the length of time between maturation and reproduction (Moorjani et al., 2016). However, although most studies use a linear regression to fit the relationship between germline mutation rate and male age at reproduction (e.g., Bergeron et al., 2020; Gao et al., 2019), two studies imply that this relationship may not be linear: in humans, the mutation rate in very young fathers appears to be disproportionally elevated as indicated by a higher number of germline mutations in short tandem repeats than predicted by a linear regression on paternal age (Forster et al., 2015). Moreover, in the first documentation of paternal age effect in humans (Kong et al., 2012) an exponential regression fitted the data at least as well as a linear regression, suggesting that the occurrence of germline mutations may not be constant over time. In addition, replication-driven mutations may be less important than assumed so far, as postmitotic neurons showed a similar mutation rate to mitotically active somatic tissues (Abascal et al., 2021). These important new findings may also explain why the estimates of paternal germline mutations are currently inconclusive even when focusing solely on humans.

In the female germline, damage-induced mutations are presumably the main source of germline mutations (Gao et al., 2019). Some underpowered studies in humans and primates found no effect of maternal age on the number of germline mutations in offspring (Besenbacher et al., 2019; Jiang et al., 2013; Venn et al., 2014; 1–32 pedigrees), but two large-scale studies report a small, linear effect of maternal age on germline mutation rate (Goldmann et al., 2016; Wong et al., 2016; > 600 pedigrees). Moreover, a recent study that re-analyzed published data from ~1,500 human pedigrees showed that an exponential regression between the number of maternally inherited germline mutations and the age of the mothers provided a significantly better fit than a linear regression (Gao et al., 2019), suggesting that damage-induced mutations may also occur in a nonlinear fashion.

The general empirical approach to studying parental age effects is based on pedigree-based data, where differences between parent and offspring genomes are assumed to be germline mutations. However, there are several shortcomings to this approach: First, in theory male-to-female ratio of germline mutations should increase with time between sexual maturation and reproduction. In most studies, this duration is roughly approximated by male age at reproduction, even though it is highly variable for example in humans (Day et al., 2016), which could obscure the relationship between the number of germline mutations and accumulation time. When maturation age and the length of time between maturation and the conception of offspring are incorporated in analyses, the model accurately predicts observed species-specific mutation rates in humans, chimpanzees *Pan troglodytes*, and owl monkeys *Aotus nancymai* (Thomas et al., 2018). Second, pedigree-based studies often use a single regression line to fit the relationship between the number of germline mutations in offspring and the age of the fathers/mothers across families, while genetic variation is largely ignored (Goldmann et al., 2021; Moorjani et al., 2016; Rahbari et al., 2016). Third, in pedigree-based studies, germline mutations are followed from one generation to the next—new variants found in offspring genomes are assumed to be germline mutations. But because germline mutations are extremely rare events (10^−10^ ~ 10^−8^ per nucleotide site per generation; Wang & Obbard, 2023), this number is expected to be very low, and the deviation of germline mutation rate from linear prediction on parental age is therefore expected to be subtle and difficult to detect if transmission is only observed from parents to offspring.

Mutation accumulation (MA) experiments offer an experimental alternative for studying germline mutation rates. Since the advent of the next-generation sequencing technology, whole-genome resequencing of MA lines (WGS-MA) has yielded fruitful results of germline mutation rates in both model (e.g., *Caenorhabditis* species, Denver et al., 2012, 2009; Baer et al., 2010; Konrad et al., 2019; Saxena et al., 2019, *Drosophila melanogaster*, Assaf et al., 2017; Huang et al., 2016; Keightley et al., 2009; Schrider et al., 2013) and non-model organisms (nematode *Pristionchus pacificus,*Weller et al., 2014, non-biting midge *Chironomus riparius,*Waldvogel & Pfenninger, 2020). When combined with experimental manipulations, WGS-MA experiments can offer insights into the determinants of germline mutation rate variation: in *Caenorhabditis elegans*, for example, germline mutation rate seems to be unrelated to the fitness of the starting genotype (Saxena et al., 2019) or population size (Konrad et al., 2019). Harnessing the MA experimental approach to study age-specific mutation rates while controlling for maturation and age at reproduction in both parents is therefore an interesting next step.

We designed a study in which (a) the age at sexual maturity and the age at reproduction of the study organism can be easily determined and manipulated, allowing an accurate measure of the length of time during which germline mutations can accumulate, (b) genetic variation in the strength of paternal and maternal age effect can be minimized, (c) the transmission of germline mutations can be followed over multiple generations. We used an MA approach in an isogenous inbred laboratory strain of the nematode *Caenorhabditis remanei* to explore age-specific germline mutation rates. *C. remanei* is dioecious and germline proliferation (mitosis) and gametogenesis (meiosis) continue in adult worms in both sexes. We created replicated MA lines of *C. remanei* by allowing the worms to reproduce at day 1 (Young T1), day 2 (Peak T2), or day 5 (Old T5) postsexual maturity, respectively. The similar rounds of mitotic and meiotic replication events in the male and female germline in juvenile and adult *Caenorhabditis* nematodes (Klass et al., 1976; Poullet et al., 2015) suggest that mutation rates are not necessarily male-driven but likely a product of mutations occurring in both sexes. Furthermore, under the assumption of linear germline mutation accumulation with age, we predicted Young T1 lines to show the lowest, Peak T2 lines an intermediate, and Old T5 lines the highest germline mutation rate. Any deviation from such a pattern suggests a nonlinear mutation rate with age.

## Materials and methods

### Study species

*C. remanei* is a free-living, dioecious nematode worm and male and female germ cells continue mitotic divisions after sexual maturity (Hubbard & Greenstein, 2005). *C. remanei* has a short lifespan of approximately 2 weeks when cultured under standard laboratory conditions (Brenner, 1974; Stiernagle, 2014). Both sexes are sexually mature and fertile upon molting into adults, and sexual maturity can be easily determined by changes in morphology (Timmermeyer et al., 2010).

### Mutation accumulation lines

We used *C. remanei* wild-type strain SP8 in this study maintained under standard lab conditions (Stiernagle, 2014). We used one male and one female from a highly inbred strain (see Supplementary materials for details) to generate replicated MA lines (SupplementaryFigure S1). Each MA line (founder generation, M0) was established by randomly selecting one virgin male and one virgin female (full siblings) from the inbred line at generation 20 (G20), resulting in all MA lines sharing an initially identical genetic background. We started MA lines for six different adult age-at-reproduction lines where offspring produced during a 24-hr window of adult age (age postsexual maturity) as follows: T1: 0–24 hr, T2: 48–72 hr, T3: 72–96 hr, T4: 96–120 hr, T5: 120–144 hr, and T6: 144–168 hr in each generation. The initial number of lines kept for each regime were: T1: 120 lines, T2: 60 lines, T3: 60 lines, T4: 60 lines, T5: 149 lines, and T6: 60 lines (see SupplementaryFigure S2). For T1 and T5, we ran two independent sets of lines at two different time points as the T5 lines went extinct after just three generations both times (see SupplementaryFigure S2) and we therefore used T5 lines from both sets for sequencing. For sequencing, we focused on Young T1, Peak T2, and Old T5 lines to cover a good range of adult age and include lines before (Young T1), during (Peak T2), and after (Old T5) the reproductive peak of the ancestral inbred lines for sequencing.

To start the next generation, we randomly collected one virgin male and one virgin female from each full-sib offspring cohort (SupplementaryFigure S1) where the time points differed between regimes during the time windows described above. The MA experiment ran for 10 generations under standard conditions (Stiernagle, 2014). MA lines for which no new offspring could be obtained to generate the next generation due to lethality, infertility, or offspring being exclusively males or females were discontinued. For both sets of Old T5 lines, this was the case already in generation 3 (M3; see SupplementaryFigure S2), and hence we collected offspring from Young T1, Peak T2, and Old T5 in generation M3 for sequencing to capture the full range of survival.

### Whole-genome resequencing

For each MA line (T1, T2, and T5), one virgin male was randomly selected from the offspring cohort of the M3 generation for whole-genome resequencing. Genomic DNA was extracted using the DNeasy Blood and Tissue Kit (Qiagen; Cat. No.: 150343) following the manufacturer’s instructions. DNA extracts were concentrated using a SpeedVac for 15 hr to reduce the volume, followed by resuspension of DNA in 4 μl of nuclease-free water. The concentration and the quality of the resuspended DNA were determined by NanoDrop spectrophotometer (ThermoFisher) and BR Qubit assay (Invitrogen). The resuspended DNA was then whole-genome amplified using the REPLI-g Single Cell Kit (Qiagen; Cat. No.: 150345) according to the protocol for amplification of genomic DNA from single cells. The REPLI-g Single Cell Kit uses multiple displacement amplification technology which enables the generation of DNA fragments without sequence bias. Briefly, 4 μl DNA resuspension was mixed with 3 μl buffer D2, and the mixture was incubated at 65 °C for 10 min. The reaction was stopped by adding 3 μl stop solution to the mixture. Then a PCR master mix (9 μl water, 29 μl REPLI-g sc reaction buffer and 2 μl REPLI-g sc DNA polymerase; a total of 40 μl for each sample) was added to the denatured DNA. Samples were incubated at 30 °C for 8 hr, and the DNA polymerase was inactivated by heating the samples for 3 min at 65 °C. The quality of amplified DNA was then determined by gel electrophoresis.

We used 1 μg DNA for library prep with the TruSeq PCRfree DNA Library Preparation Kit (Illumina Inc.; Cat. No.: FC-121-3001/3002). Multiplexed DNA libraries were paired-end sequenced using Illumina HiSeqX instrument and v2.5 chemistry, with a read length of 150bp at the SNP&SEQ Technology Platform in Uppsala.

### Data processing

Raw reads in fastq format were individually adapter- and quality-trimmed using fastp/0.23.2 (Chen et al., 2018) and mapped against the latest *C. remanei* reference genome (ASM164373v4; GeneBank Accession No.: GCA_010183535.1) using mem, BWA/0.7.17 (Li, 2013). Aligned reads were then sorted (using samtools/1.16.1 sort), indexed (using samtools/1.16.1 index) (Danecek et al., 2021), and duplicates marked (using Picard/2.24.1 MarkDuplicates; http://broadinstitute.github.io/picard/). Marked duplicated reads were ignored in downstream processes. The resultant bam files were indexed (using samtools/1.16.1 index) and then used for mutation calling.

### Mutation calling

We focused on heterozygous point mutations (single-nucleotide mutations, single-nucleotide polymorphisms [SNPs]) and used two independent approaches to call candidate mutations. First, a consensus approach using GATK’s HaplotypeCaller, GenomicsDBImport, and GenotypeGVCFs tools (Poplin et al., 2017), and second, a probabilistic approach using the accuMUlate model (Winter et al., 2018). Both are explained in detail in the Supplementary methods.

### Manual inspection

Because the two mutation calling approaches use different algorithms to identify mutations, we performed manual curation to detect and remove false-positive calls (Bergeron et al., 2022). Specifically, we manually inspected the alignment reads surrounding each candidate SNP using Integrated Genome Viewer (Thorvaldsdottir et al., 2013). Following Keightley et al. (2014) and (Sasani et al., 2019), we excluded candidate SNPs that are likely to be false positives. See Supplementary Figures S3 and S4 for exemplary screenshots (SupplementaryFigure S3: consensus approach; SupplementaryFigure S4: probabilistic approach); see Supplementary materials for more details on acceptance criteria. Chi-squared tests were used to test if the acceptance rate differed between regimes.

### Calculation of mutation rates

Line-specific, per-site, per-generation mutation rates for a diploid genome were calculated for each of the 18 MA lines using the equation *μ = m/*(L*n*T) (Denver et al., 2012), where *μ* is the mutation rate per nucleotide site per generation, *m* is the number of line-specific mutations, *L* is the number of MA lines (*L* = 1 for line-specific mutation rate), *n* is the number of nucleotide sites accessible for mutation calling (i.e., the number of callable sites, see below), and *T* is the number of generations of mutation accumulation (*T* = 3 in our study). We calculated *n* for each regime as the number of sites where all MA lines in the same regime were genotyped.

### Mutation spectrum, and the number of transitions and transversions

We analyzed the mutation spectrum by considering the frequencies of the six base-substitution types (A:T->G:C, G:C->A:T, A:T->C:G, G:C->T:A, A:T->T:A, G:C->C:G). Chi-squared tests were used to test if their relative frequencies differed between regimes. For chi-squared tests, *p* values were simulated based on 10,000 replicates.

The number of type-specific mutations was pooled into transitions (A:T->G:C, G:C->A:T) or transversions (A:T->C:G, G:C->T:A, A:T->T:A, G:C->C:G) for each regime. Similar to the above, we used chi-squared tests to test if the numbers of transitions and transversions differed between regimes. For chi-squared tests, *p* values were simulated based on 10,000 replicates.

### Prediction of mutational effects

Because the reference assembly we used in our analyses (ASM164373v4) was not yet annotated, we identified the corresponding nucleotide site in an annotated *C. remanei* assembly (CRPX506) for the candidate SNPs and used these for variant effect prediction. Specifically, we used the sequences flanking the candidate SNPs (±200bp) in ASM164373v4 as query sequences and performed standard nucleotide BLAST (blastn) against the CRPX506 assembly (Altschul et al., 1990). For most of the candidate SNPs, this resulted in a single hit with high similarity scores, suggesting the ambiguity in the corresponding genomic locations was minimal. The corresponding genomic locations in the CRPX506 assembly for the candidate SNPs were then extracted. The genomic locations, along with the nucleotide changes for each SNP, were used for variant effect prediction.

We used the variant effect prediction tool available on WormBase to categorize the potential effects of the mutations in the MA lines. Goodness-of-fit tests were used to test if the relative frequencies in each category differ between MA regimes.

### Statistical analyses

To test whether the germline mutation rate increases with increasing length of accumulation time, we fitted two separate generalized linear models to compare the germline mutation rates between the regimes. First, a generalized linear model with a Poisson error distribution was fitted with the number of mutations included as the response variable, the number of callable sites included as weight, and the MA regime (Young T1, Peak T2, or Old T5) included as a fixed effect. Second, a generalized linear model with a binomial error distribution was used with the number of mutated sites and the number of nonmutated sites (i.e., the number of callable sites minus the number of mutations) fitted as the response variable, and MA regime (Young T1, Peak T2, or Old T5) included as a fixed effect. All statistical analyses were conducted using the R software (R Core Team, 2020) and the RStudio software (RStudio Team, 2020) and the packages *dplyr* (Wickham et al., 2023), *tidyr* (Wickham & Henry, 2020), *lme4* (Bates et al., 2015), *car* (Fox & Weisberg, 2019), *ggplot2* (Wickham, 2016), *gridExtra* (Auguie, 2015), and *wesanderson* (https://github.com/karthik/wesanderson).

## Results

### Line survival

**Figure 1.**
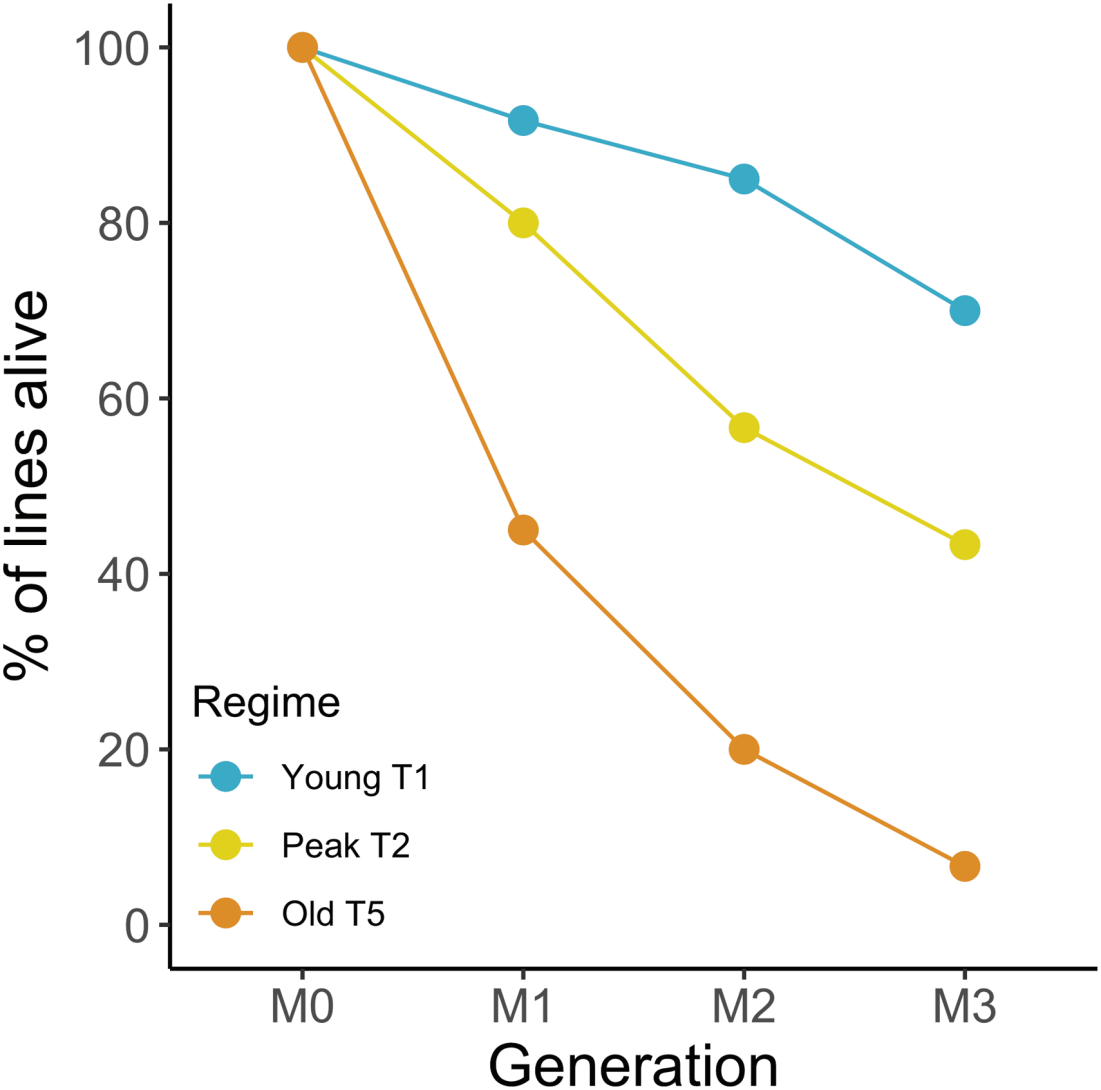
Line survival rate across generations shown for the Young T1 (orange), Peak T2 (yellow), and Old T5 (blue) mutation accumulation (MA) regimes.

Although some MA lines were kept for up to 10 generations (Young T1 and Peak T2) most MA lines involving older age-at-reproduction regimes had to be stopped earlier (SupplementaryFigure S2). For Old T5 lines, both sets ended already in generation M3 (Figure 1). The parental age at reproduction strongly affected the survivorship across the three focal regimes YoungT1, Peak T2, and Old T5 (Figure 1).

### Mutation accumulation

After three generations of mutation accumulation, the genome derived from one male from each of the six lines for each three MA regimes (18 males in total) was sequenced. Samples were sequenced to an average of 66.00X coverage depth per individual, with an average of 68.50X in Young T1 lines, 76.59X in Peak T2 lines, and 52.92X in Old T5 lines (see SupplementaryTable S1 for the mean coverage depth of each line). Due to technical issues, the coverage depth of MA line T1–57 of the Young T1 regime (15.71X) is significantly lower than the rest of the Young T1 lines. To determine whether this could compromise our power in detecting mutations, we performed a preliminary analysis with T1–57 either included or excluded. We found no evidence that including or excluding T1–57 affected the outcomes. Therefore, we decided to include T1–57 in our analysis. The callable sites represent on average 74.20% of the ASM164373v4 genome (Young T1 lines: 72.75%, Peak T2 lines: 75.36%, and Old T5 lines: 74.50%; see SupplementaryTable S1).

The consensus approach and the probabilistic approach initially identified 940 and 343 candidate mutations, respectively. After manual inspection, we accepted 175 (consensus approach) and 319 (probabilistic approach) candidate mutations, with 37 mutations shared between these two approaches (see SupplementaryTable S2 for details; SupplementaryFigure S5). The acceptance rate did not differ between regimes (consensus approach: *X*^*2*^ = 0.04, *df* = 2, *p* = 0.98; probabilistic approach: *X*^*2*^ =0.01, *df* = 2, *p* = 1.00). These 37 mutations were used for further statistical analyses and comparisons between regimes.

The results of analyses reported here were based on a union cohort (a commonly used approach; see Assaf et al., 2017; Katju et al., 2022; Konrad et al., 2019; Pfenninger et al., 2022), and the results of analyses based on the respective cohorts of accepted candidates identified by the two approaches are reported in the Supplementary materials.

### Germline mutation rates and the lengths of mutation accumulation time

**Figure 2.**
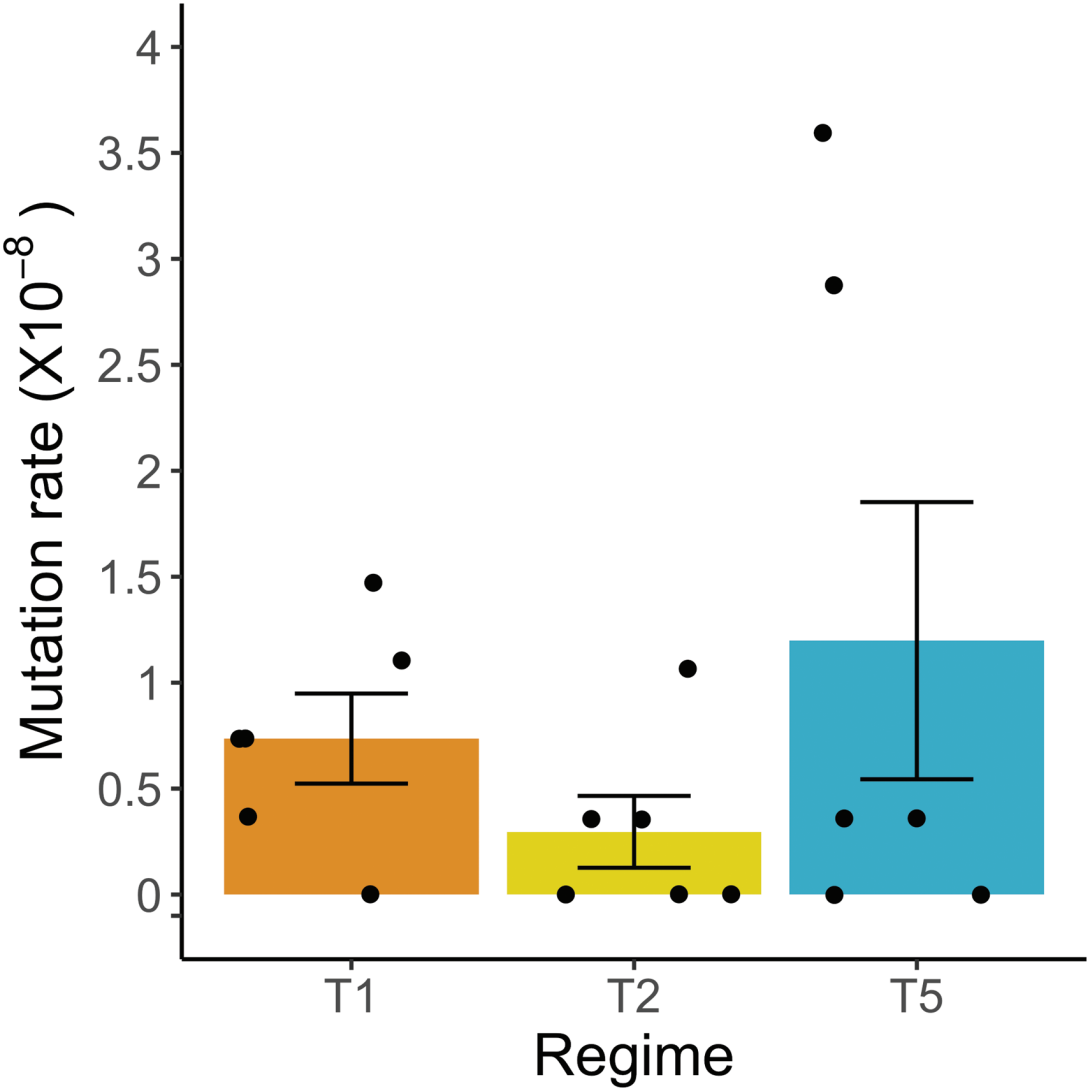
Mean heterozygous point mutation rates of *C. remanei* mutation accumulation (MA) lines in three treatment regimes: Young T1 (orange), short mutation accumulation time; Peak T2 (yellow), intermediate mutation accumulation time; and Old T5 (blue), long mutation accumulation time. *N* = 6 lines per regime (represented by black points). Solid vertical lines represent standard errors (s.e.).

Of the 37 accepted candidates, 12 were found in Young T1 lines (2.00 ± 0.58 per line), 5 in Peak T2 lines (0.83 ± 0.48 per line), and 20 in T5 lines (3.33 ± 1.82 per line), respectively (Table 1 and SupplementaryTable S2). We calculated the per-generation base-substitution mutation rate for each MA line by dividing the number of line-specific mutations by the product of the number of callable sites and the number of generations of mutation accumulation. The mutation rates range from 0 to 3.60 × 10^−8^ across the lines (see SupplementaryTable S4 for details). Mutation accumulation differed significantly between regimes both for absolute numbers of mutations (generalized linear model with Poisson error distribution: *X*^*2*^ = 759,046,551, *df* = 2, *p* < .001) and for relative number of mutations (generalized linear model with binomial error distribution: *X*^*2*^ = 8.16, *df* = 2, *p* < .05) with the Peak T2 regime showing a significantly lower mutation rate than both Young T1 and Old T5 regimes (average mutation rates per nucleotide per generation: Young T1 regime: 0.74 (±0.21) × 10^−8^; Peak T2 regime: 0.30 (±0.17) × 10^−8^; Old T5 regime: 1.20 (±0.65) × 10^−8^; Tables 1 and 2; Figure 2).

**Table 1.** Mean number (± standard error, s.e.) of mutations, callable sites, mutation rate (μ), transitions, and transversions for each regime.

	Young T1	Peak T2	Old T5
No. of mutations	2.00 (±0.58)	0.83 (±0.48)	3.33 (±1.82)
Callable sites	90,566,554	93,817,854	92,742,405
μ (×10^−8^)	0.74 (±0.21)	0.30 (±0.17)	1.20 (±0.65)
Transitions	2.00 (±0.58)	1.67 (±0.48)	3.17 (±1.70)
Transversions	0.00	0.00	0.17 (±0.17)

**Table 2.** Mean number of mutations, callable sites, mutation rate (μ), transitions, and transversions by each approach for each regime.

Approach	Consensus approach			Probabilistic approach		
Regime	Young T1	Peak T2	Old T5	Young T1	Peak T2	Old T5
No. of mutations	11.50 (±2.87)	5.75 (±1.03)	13.83 (±6.35)	25.67 (±9.07)	7.17 (±3.11)	20.33 (±9.10)
Callable sites	90,566,554	93,817,854	92,742,405	90,566,554	93,817,854	92,742,405
μ (×10^−8^)	4.23 (±1.06)	1.36 (±0.49)	4.97 (±2.28)	9.45 (±3.34)	2.55 (±1.11)	7.31 (±3.27)
Transitions	10.83 (±2.60)	3.67 (±1.33)	12.83 (±6.02)	22.83 (±8.11)	6.50 (±3.12)	18.17 (±7.79)
Transversions	0.67 (±0.33)	0.17 (±0.17)	1.00 (±0.37)	2.83.83 (±1.01)	0.67 (±0.33)	2.17 (±1.38)

### Mutation spectrum, transition, and transversion

**Figure 3.**
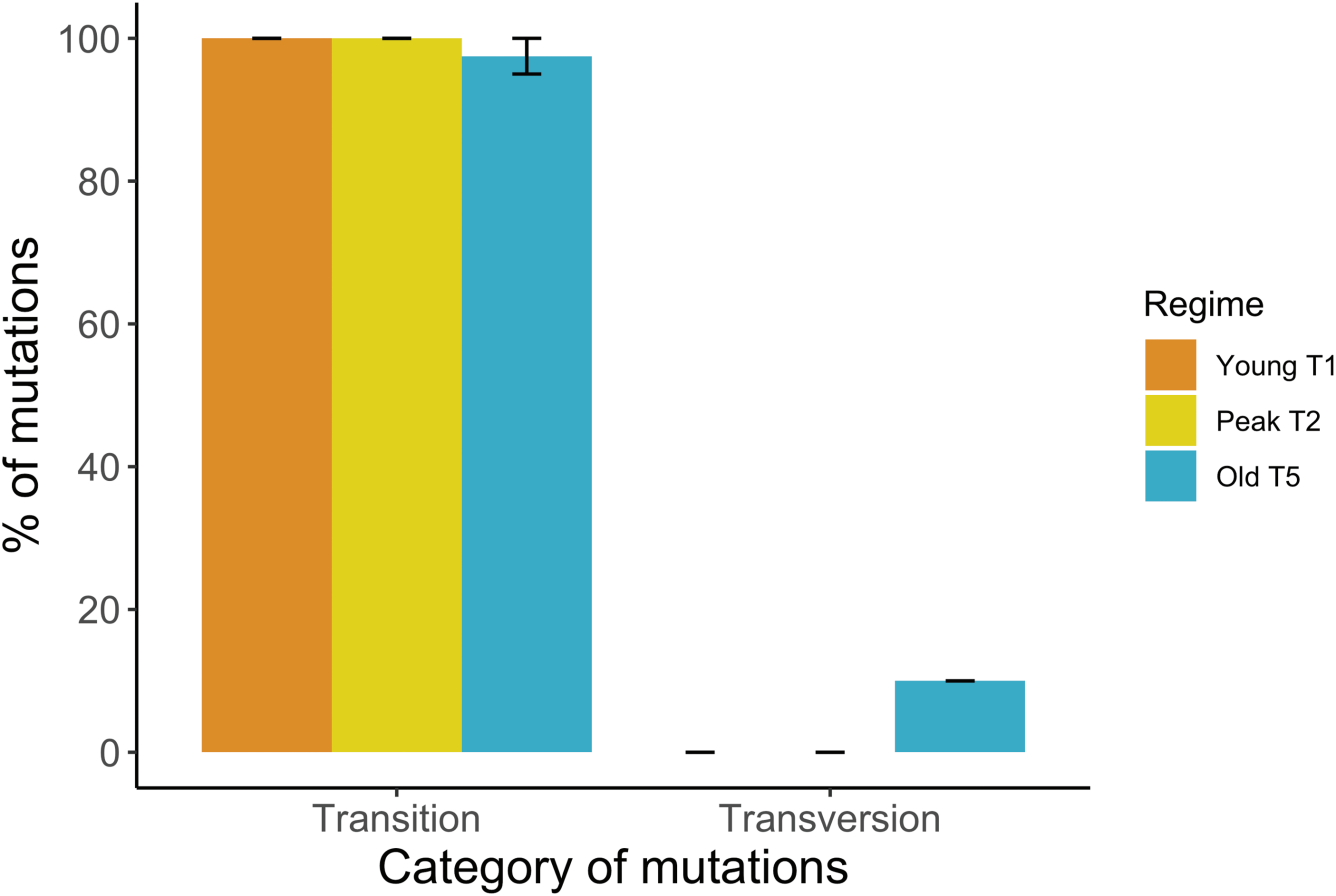
Distribution of the six types of single-nucleotide mutations of the mutation accumulation (MA) lines in three treatment regimes: Young T1 (orange), short mutation accumulation time; Peak T2 (yellow), intermediate mutation accumulation time; and Old T5 (blue), long mutation accumulation time. *N* = 6 lines per regime. Bars show mean % of mutations across the six lines per regime with standard error (s.e.) bars.

We did not observe any significant differences in mutation spectrum among regimes (chi-square test: *X*^*2*^ = 3.67, *p* = .47; SupplementaryTable S4). For Young T1 and Old T5 regimes, significantly more transitions occurred than transversions; whereas the significance in the Peak T2 regime is marginal, possibly due to the small number of total mutations (Young T1:12 transitions and 0 transversion, *X*^*2*^ = 12, *p* < .001; Peak T2: 5 transitions and 0 transversion, *X*^*2*^ = 5, *p* = .06; Old T5: 19 transitions and 1 transversion, *X*^*2*^ = 16.2, *p* < .001; Tables 1 and 2; Figure 3). The results were very similar when comparing the two mutation calling methods (SupplementaryFigure S6).

### Prediction of mutational effects

Not all variant groups had high enough sample sizes for statistical evaluation and we only included those that did. While moderate impact variants did not differ among regimes (*N* = 9; *X*^*2*^ = 0.67, *p* = .92), we found significantly more modifier variants in Young T1 and Old T5 worms than in Peak T2 worms (*N* = 84; *X*^*2*^ = 21.5, *p* < .001), and more low impact variants in Old T5 lines than in Young T1 lines and Peak T2, albeit the significance was marginal (*N* = 8; *X*^*2*^ = 6.25, *p* = .06). Notably, no high impact variants occurred in Peak T2 regime and the only two were found exclusively in Young T1 worms.

**Figure 4.**
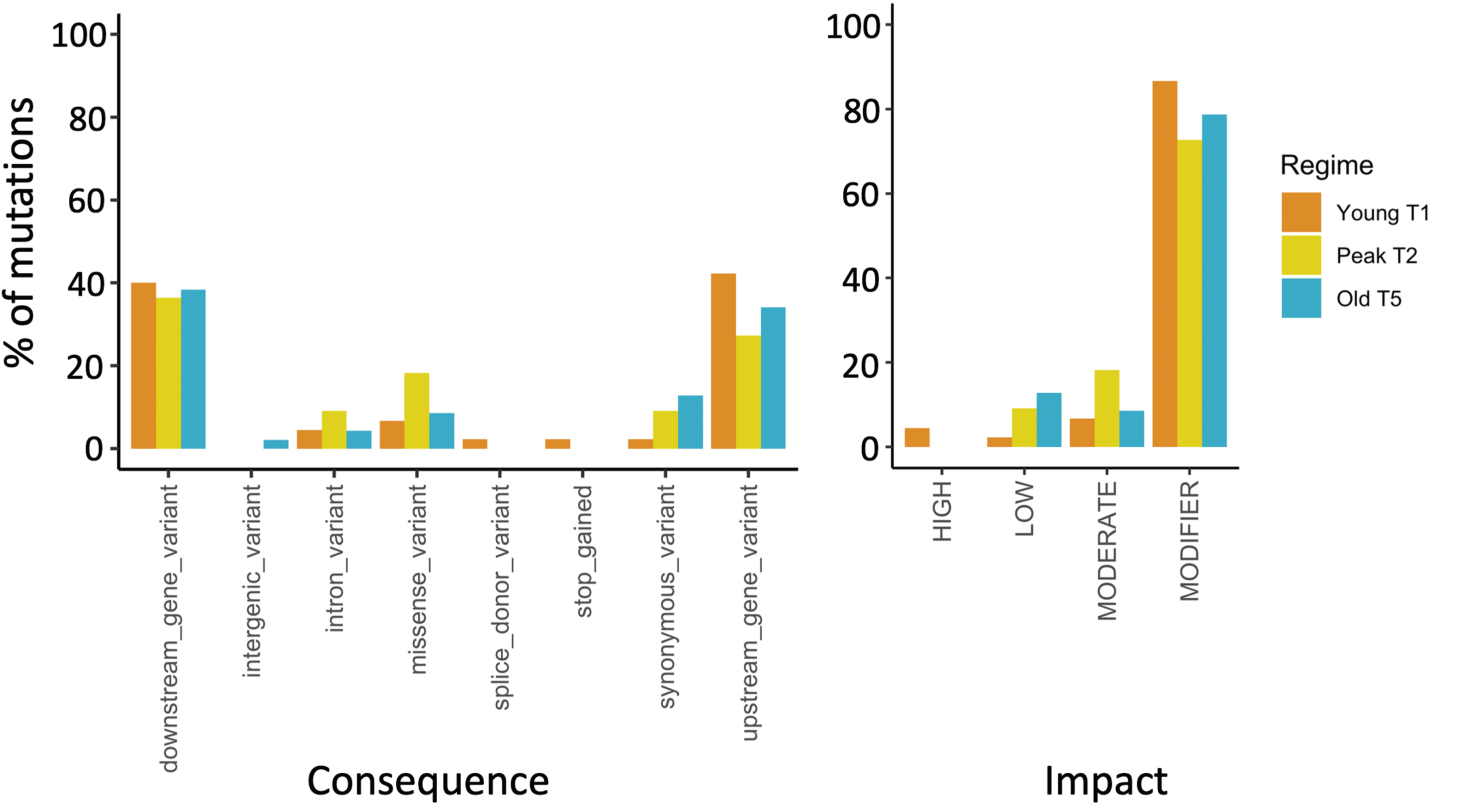
Distribution of the consequences (a) and impact (b) of single-nucleotide mutations of the MA lines in three treatment regimes: Young T1 (orange), very young worms and short mutation accumulation time; Peak T2 (yellow), worms at the peak of their reproductive fitness and intermediate mutation accumulation time; and Old T5 (blue), old worms and long mutation accumulation time (*N* = 6 males from six different lines per regime).

We found no significant difference among the regimes for intron variants (*N* = 5; *X*^*2*^ = 0.4, *p* = 1) and missense variants (*N* = 9; *X*^*2*^ = 0.67, *p* = 0.91), but downstream gene variants (*N* = 40; *X*^*2*^ = 9.8, *p* < .01) and upstream gene variants (*N* = 38; *X*^*2*^ = 11.42, *p* < .01) were significantly more frequent in Young T1 and Old T5 worms than in Peak T2 worms. For synonymous variants, more variants were found in Old T5 lines than in Young T1 and Peak T2 lines with marginal significance (*N* = 8; *X*^*2*^ = 6.25, *p* = .06). Stop-gained and splice donor variants (one variant each) were rare and were only found in Young T1 worms, and the only intergenic variant was found in an Old T5 line (Figure 4; Table 4).

**Table 4.** Number of the consequences and impact of single-nucleotide mutations of the MA lines in three treatment regimes.

		Young T1	Peak T2	Old T5
Consequence	downstream_gene_variant	18	4	18
	intergenic_variant	0	0	1
	intron_variant	2	1	2
	missense_variant	3	2	4
	splice_donor_variant	1	0	0
	stop_gained	1	0	0
	synonymous_variant	1	1	6
	upstream_gene_variant	19	3	16
Impact	HIGH	2	0	0
	LOW	1	1	6
	MODERATE	3	2	4
	MODIFIER	39	8	37

## Discussion

Peak T2 lines reproducing on day 2 of adulthood had accumulated a lower overall number of germline mutations than the Young T1 and Old T5 lines, which both showed similar and higher germline mutation rates (Figure 2). Interestingly, this pattern varied for different groups of variants and some groups such as modifiers and up- and downstream gene variants were less frequent in Peak T2 worms (Figure 4; Table 4). Survival was directly associated with age at reproduction and Young T1 lines showed the highest survival rates, whereas Old T5 lines showed the lowest survival rates and went extinct very rapidly (Figure 1; also see SupplementaryFigure S2). One plausible explanation for our findings is that when the inbred line was generated, the worms were bred on day 2 of adulthood, and hence germline-specific genome maintenance and repair mechanisms have evolved to be optimal during that age. Worms did not contribute to the next generation during the initial inbreeding phase at Young and Old ages and hence germline maintenance and repair at these ages were under relaxed selection. We discuss our findings and interpretation in a broader context below.

### Germline mutation rate and the age at reproduction

The mean germline mutation rate for the T2 regime of 0.30 × 10^−8^ (±0.17) lies within the typical range of germline mutation rate estimates of *C. elegans* and *C. briggsae* (0.1–0.3 × 10^−8^; Denver et al., 2009, 2012; Konrad et al., 2019; Saxena et al., 2019; Table 3). In all these previous studies, worms were invariably propagated at a 4-day interval during the mutation accumulation experiments where every 4 days, a single immature L4-stage hermaphrodite was transferred to establish a new population (*C. elegans* and *C. briggsae* are both primarily hermaphroditic selfers, and L4 is the last immature stage). Because it takes approximately 2 days for an egg to develop into an L4 stage larva (Sutphin & Kaeberlein, 2009), propagating the MA lines every 4 days would mean that the average parental age at reproduction was 2 days—which is in line with our T2 Peak regime, where worms were propagated at 24–48 hr postsexual maturity (see SupplementaryFigure S1). Our estimates for T1 Young and T5 Old regimes (0.74 × 10^−8^ (±0.21) for T1 and 1.20 × 10^−8^ (±0.65) for T5) were higher than reported for other nematode species (Table 3). However, with the comparability of the T2 regime and *C. elegans* and *C. briggsae* in mind, it is intuitive that the elevated germline mutation rates in these two regimes stem propagation outside their reproductive peak.

**Table 3.** Summary of mutation rate (μ), within-species fold change in mutation rate, and transition-to-transversion ratio in nematodes and other invertebrates.

Group	Species	Genotype/treatment	μ (×10^−8^)^a^	Fold difference in μ	Ts:Tv	Reference
Nematode	*Caenorhabditis briggsae*	Wild-type strain HK104	0.10 ± 0.07 − 0.14 ± 0.03	2	0.72	Denver et al., 2012
		Wild-type strain PB800	0.10 ± 0.02 − 0.20 ± 0.04		1.14	Denver et al., 2012
Nematode	*Caenorhabditis elegans*	Wild-type strain PB306	0.13 ± 0.03 − 0.21 ± 0.05	3.38	1.06	Denver et al., 2012
		Wild-type strain N2	0.08 ± 0.04 − 0.18 ± 0.05		0.64	Denver et al., 2012
		Wild-type strain N2	0.27 ± 0.04		0.19–0.79	Denver et al., 2009
		Wild-type strain N2	0.26 ± 0.01		0.74	Saxena et al., 2019
		Wild-type strain N2	0.18		0.36–1.04	Konrad et al., 2019
Nematode	*Caenorhabditis remanei*	Wild-type strain SP8	0.30 ± 0.17 − 1.20 ± 0.65	10	36	This study
Nematode	*Pristionchus pacificus*	Wild-type strain PS312	0.14 − 0.26	1.86	3.9	Weller et al., 2014
Water flea	*Daphnia magna*	Wild-type Finland population	2.26 − 6.70	9.3	1.32–1.78	Ho et al., 2020
		Wild-type Israel population	0.72 − 0.92		1.00–2.64	Ho et al., 2020
Water flea	*Daphnia pulex*	Asexual genotype	0.72 ± 0.33	1.6	1.58	Keith et al., 2016
		Cyclically parthenogenetic genotype	0.45 ± 0.02			Keith et al., 2016

^a^Standard errors are provided in parenthesis (±s.e.) whenever available.

### Age-specific mutation rate and selection shadow

Although parental age effects on germline mutation rate have been demonstrated as a positive correlation between the number of mutations in offspring and the reproductive age of fathers or mothers (Bergeron et al., 2020; Gao et al., 2019), the underlying mechanisms leading to increased occurrence of germline mutations with increasing parental age are still debated. In some studies, the mutation rate appears to show a linear increase while others are reporting an exponential increase with parental age. This may be partly due to the imprecise approximation of age at reproduction as a direct measurement of the length of mutation accumulation time due to variation in the onset of maturation among individuals (Thomas et al., 2018). Individuals showing higher germline mutation rates at a given age may have matured at an earlier age. In our experiment, germline mutations were allowed to accumulate in a highly standardized manner in the three experimental regimes and the observed germline mutation rates in the three regimes directly reflect the ontogenetic differences in germline mutation rates. The increased mutation rate in Old worms from the T5 regime is expected under a scenario of increasing germline mutation rate with age. In contrast, the higher mutation rate in T1 Young worms compared to T2 Peak worms indicates an age-specific decrease in germline mutation rate. This pattern does not fit the prediction of a linear increase in germline mutation rate with age.

In *C. remanei*, mitotic germline proliferation occurs during the early larval stages from two cells at hatching to ~400 cells in stage 4 larvae (L4). Meiotic entry begins already in stage 3 larvae (L3; Roy et al., 2016), and young adults are immediately fertile upon emergence. The first gametes used by young adults are therefore likely to have been generated prior to sexual maturity. The higher mutation rate in the Young T1 regime can thus be interpreted as a higher mutation rate in the presexual maturity phase. This scenario is consistent with a recent study in humans, which used multisibling families to identify the timing of germline mutations during ontogenesis and suggested that per cell division mutation rate is higher during early embryogenesis and differentiation of primordial cells of the parents, and is then considerably reduced in male germline after maturation (Lindsay et al., 2019; Rahbari et al., 2016). For this reason, a decrease in germline mutations with age is possible when germline stem cells are lost and replaced (Wallenfang et al., 2006).

During the generation of the inbred lines forming the ancestors for the MA lines, worms were generally transferred for breeding on day 2 of adulthood for 20 generations. This means that selection for reduced mutation and increased germline genome repair and maintenance was strongest during this window of time which was reflected in our day 2, Peak T2 regime. Young T1 and Old T5 animals on the other hand have been under less stringent selection during the inbreeding stage of the experiment, which could explain the higher mutation accumulation rate in these two regimes. Furthermore, inbreeding depression increases with age (Charlesworth & Hughes, 1996), and hence breeding in late life means reduced reproductive fitness which can contribute to the increased line extinction rates in Old T5 lines (Figure 1). In fact, the “selection shadow” during the 20 generations of the inbreeding stage of the experiment allowed for the accumulation of mutations with deleterious effects late in life, as old worms never reproduced (Medawar, 1952). Such mutations may explain the high extinction rates in the Old T5 lines due to the low reproductive fitness in 5-day-old worms. In addition, these age-specific mutations may also affect the overall condition of the worms, thereby reducing the germline repair and maintenance mechanisms in old age which led to the observed increased mutation rate.

### Paternal vs maternal mutation accumulation

In many taxa, male germline proliferation and spermatogenesis continue throughout life while the female germline develops during early embryo development and oogenesis halts during the second meiotic division before the female reaches sexual maturity. As a consequence, the number of replication-driven mutations inherited by offspring is expected to increase with the age of the father but not with the age of the mother (Crow, 2000; Li et al., 1996a) and that germline mutations are directly correlated with paternal age (Crow, 2000; Drost & Lee, 1995). A paternal age effect on mutation rate has been reported in humans (Bergeron et al., 2023; Besenbacher et al., 2016; Francioli et al., 2015; Goldmann et al., 2016; Jiang et al., 2013; Jónsson et al., 2017; Kong et al., 2012; Lindsay et al., 2019; Michaelson et al., 2012; Rahbari et al., 2016; Wang et al., 2022; Wong et al., 2016), other mammals (e.g., mouse, Lindsay et al., 2019; cat, Wang et al., 2022), and other amniotes (de Manuel et al., 2022). In all cases, a positive correlation was found between the number of mutations observed in the offspring and the age of the father at reproduction. Contrary to the studies mentioned above, in *C. remanei*, cell line mitotic proliferation (L1–L3) and meiotic divisions (L4) occur at similar time points in both males and females and start during the various larval stages and continue during the adult stage (Kimble & Hirsh, 1979; Miller et al., 2001). Our results therefore are not a clear effect of male age but likely the combined effects of male and female germline proliferation and gametogenesis.

### Types of mutations

Germline mutations can arise from (a) the misincorporation of nucleotides on the newly synthesized DNA strand during replication (replication-driven), (b) from unrepaired damage of the DNA template or free nucleotides between replication cycles (damage-induced), or from the interaction of the two (Poulos et al., 2017). Although the relative importance of the two sources is currently unclear, replication-driven mutations are generally assumed to be the primary source of germline mutations (Crow, 2000; Drost & Lee, 1995; Li et al., 1996b). This assumption has led to the idea of male-driven mutation rates. However, our results suggest that the importance of the two mechanisms likely depends on the ontogenetic stage of the organism and varies throughout life. The maintenance and repair of the germline can be costly (Chen et al., 2020; Maklakov & Immler, 2016), and depending on the importance, organisms may downregulate these mechanisms during life stages where these are less relevant and upregulate them during peak reproductive stages.

The up- and downregulation of the repair mechanisms may also be reflected in our findings that different types of mutations vary in abundance across the different regimes, and sometimes frequencies vary in opposite directions. Not surprisingly, mutation variants with strong effects were low or absent across all regimes due to their lethal effects and resulting removal from the lines across generations. Modifier variants were also significantly less frequent in Peak T2 lines (Figure 4; Table 4), although their impact on gene expression may vary and it is currently unclear whether these are mild mutations or mutations with stronger but potentially beneficial effects. In contrast, synonymous variants and low-impact mutations were highest in Old T5 lines which may reflect age-related accumulation at no phenotypic cost (Figure 4a; Table 4). These results confirm further that the mutation rates estimated are both replication and damage-driven, depending on the ontogenetic stage. In Young T1 lines, the higher mutation rate may well be the result of replication-driven mutations and reduced germline maintenance and repair, whereas in T5 lines, the increased mutation rate may be the result of both, although damage-driven mechanisms are likely to play a more important role.

The Ts:Tv in our results is relatively high compared to other species (Table 3). The mutational bias hypothesis assumes that the rates at which transitions and transversions arise depend on the environment (Gojobori et al., 1982; Vogel & Kopun, 1977). This hypothesis predicts that Ts:Tv ratio likely differs between MA lines and natural isolates of the same species due to changes in the lab environment. This prediction is supported by observations in *C. elegans* and *C. briggsae* where Ts:Tv ratios were lower in MA lines than in natural isolates (Denver et al., 2012; Saxena et al., 2018). More intriguingly, if Ts:Tv ratio is determined by the rearing environment, Ts:Tv ratio would be expected to differ between laboratories. Indeed, in *C. elegans* N2 strains, where Ts:Tv ratios are available from multiple studies, Ts:Tv ratios did differ between laboratories (see Table 3). We therefore speculate that this was the likely explanation for the high Ts:Tv ratio in our study. Future studies on germline mutation rates across *Caenorhabditis* species should ideally involve a range of methodologies (such as pedigree-based and mutation accumulation) to provide further insights.

### Wider implications

Understanding how germline mutations accumulate over time as an individual develops and ages has far-reaching implications, particularly in understanding the variation in substitution rate, and consequently, in setting the evolutionary clock, to which we can relate genetic data to infer the timescales of evolutionary events. Observations from studies of mammalian phylogenies have shown that species with longer generation times (i.e., average age of reproduction) generally have lower substitution rates (Ellsworth et al., 1996; Thomas et al., 2010). The generation-time effect hypothesis proposes that this trend is due to that organisms with longer generation time accumulate mutations at a slower rate, assuming an increase in generation time decreases the number of germ cell divisions per unit time. As a species evolves, its life history changes, and the average age of reproduction may shift towards younger or older ages. The generation-time effect hypothesis predicts that a shift in age of reproduction towards older age should result in a slower substitution rate (Lehtonen & Lanfear, 2014); however, this effect is expected to be mitigated, at least partially, if older parents pass more mutations to their offspring than younger parents.

## Supplementary Material

qrad052_suppl_Supplementary_Figures_S1-S6_Tables_S1-S4

## Data Availability

The datasets and the scripts to analyze the data, as well as IGV screenshots, are available on GitHub: https://github.com/hweiyenchen/Nematode_ASM164373v4/.
