# Supplementary material for: Germline mutation rate is elevated in young and old parents in Caenorhabditis remanei
Source: Evol Lett. 2023 Oct 17;7(6):478–89. doi: 10.1093/evlett/qrad052 (PMC10692996; doi:10.1093/evlett/qrad052)
Supplement: qrad052_suppl_Supplementary_Figures_S1-S6_Tables_S1-S4 [file qrad052_suppl_supplementary_figures_s1-s6_tables_s1-s4.docx]

**Supplementary Material for**

**Germline mutation rate is elevated in young and old parents *in Caenorhabditis remanei***

Hwei-yen Chen; Therese Krieg; Brian Mautz; Cécile Jolly; Douglas Scofield; Alexei A. Maklakov; Simone Immler

**Supplementary Methods**

*Inbreeding regime*

The progenitor inbred line for the mutation accumulation (MA) lines was developed in the lab by full-sib inbreeding for 20 generations under standard lab conditions (see Figure S1 for schematic illustration of the experimental procedure). Specifically, the inbred line (founder generation, G0) was established by randomly selecting one virgin male and one virgin female from a wild-type, outbred base population and allowing them to reproduce for two days after they reached sexual maturity. The inbred line was propagated by randomly selecting one virgin male and one virgin female (who were full siblings) from the offspring cohort approximately ~72 hours after the parents reached sexual maturity each generation for 20 generations (Figure S1).

*Mutation calling*

Consensus approach

To identify germline mutations in our MA lines we took advantage of the fact that all MA lines were direct descendants of two progenitor individuals, and assumed that 1) the genotype shared across all or most lines was the ‘null’ genotype in the progenitors, and 2) unique SNPs discovered in only one MA line that were different from the allele shared by the other lines were assumed to be a line-specific germline mutation that occurred during the mutation accumulation process. Because the progenitors had been through 20 generations of inbreeding leading to very high levels of homozygosity, we focused on heterozygous loci that were likely to have arisen from segregating variants. Specifically, raw variants were first identified for each individual line using GATK/3.7 HaplotypeCaller in GVCF model. The resultant gvcf files of all lines of the same MA regime were then merged using GATK/3.8-0 GenotypeGVCFs, producing a combined vcf file containing all raw variants of the focal MA regime. The resultant vcf files were then processed and variants that fulfilled the follow criteria were included:

1. Single-nucleotide polymorphisms (SNPs); insertions/deletions were excluded;
2. Biallelic variants (sites with more than one alternate allele were excluded);
3. Variants with coverage between 0.5X and 2X of the mean coverage of the mutant MA line;
4. Variants of high quality and low strand bias (QD<2.0||FS>60.000||MQ<40.00||MQRankSum<-12.5||ReadPosRankSum<-8.0||SOR>4.0|| GQ<30.0 were excluded);
5. Singletons (SNPs found in more than one MA line of the same MA regime were excluded);
6. Variants that were heterozygous for the reference and alternate allele in the mutant MA line, and homozygous for the reference allele in the remaining MA lines of the same MA regime;
7. Variants that were heterozygous with the alternate allele ratio between 0.25 and 0.75 in the focal MA line;
8. Variants where the mutant (alternate) allele was only found in the mutant MA line, and no reads with the mutant (alternate) allele was found in all other MA lines of the same MA regime.

Probabilistic approach

We ran accuMUlate separately for each regime following the instructions to identify variant sites with a mutation probability > 0.1. We used the following parameter values: Base quality cutoff (-q) = 13; mapping quality cutoff (-m) = 13; mutation probability cutoff (-p) = 0.10000000000000001; experiment-long mutation rate (--mu) = 0.00000001; Probability of sequencing error (--seq-erro) = 0.01; theta (--theta) = 0.0001; Over-dispersion for haploid sequencing (--phi-haploid) = 0.001; Over-dispersion for diploid sequencing (--phi-diploid) = 0.001; Ploidy of ancestor (--ploidy-ancestor) = 2; Ploidy of descendant (--ploidy-descendant) = 2. The resultant tsv files, which contained all raw variants of all lines of the same MA regime called by accuMUlate, were than further processed and variants that fulfilled the follow criteria were included (see Figure S4 as example):

1. Estimated probability of at least one mutation at the site = 1;
2. Estimated probability of exactly one mutation at the site = 1;
3. Probability of the mutation direction being correct = 1;
4. Homozygous to heterozygous mutations;
5. Number of reads for the mutant (alternate) allele in non-mutant samples = 0;
6. Allelic ratio for the mutant (alternate) allele in the mutant MA line is between 0.25 and 0.75;
7. AD test statistic for mapping quality difference between mutant (alternate) and non-mutant (reference) alleles =< 1.95;
8. AD test statistic for insert size difference between inferred insert size between mutant (alternate) and non-mutant (reference) alleles =< 1.95;
9. *P*-value from Fisher's exact test of strand bias between mutant (alternate) and non-mutant (reference) alleles is > 0.05;
10. *P*-value from Fisher's exact test of pair-mapping rate difference between mutant (alternate) and non-mutant (reference) alleles is > 0.05.

*Manual inspection*

Manual inspection was performed for each regime separately. To perform manual inspection, the reference assembly ASM164373v4 was first loaded into IGV. The bam files of all MA lines of the focal regime and the vcf file for the focal regime were loaded into IGV with each bam and the vcf file representing a separate horizontal track, and their respective IDs were shown in the left panel and their genotype and alignment shown in the right panel (Figure S3 & S4). Specifically, the topmost track represented the vcf file, where each row corresponded to a MA line (whose ID was shown on the left panel) and each column in the right panel corresponded to a nucleotide site, such that each cell in the right panel indicated the genotype of the MA line at the nucleotide site. Grey cells meant the genotype for the MA line at the site was homozygous for the reference allele (which was indicated in the lowermost track); blue cells meant that the genotype for the MA line at the site was not homozygous for the reference allele. Track 2 to 7 represent an MA line. Within each track, the upper, vertical bars in the right panel showed the coverage and the allelic fraction for the nucleotide found at the site: grey bars mean that all reads at the site had the reference allele, whereas colored bars (blue, green, red or brown) mean that at least some reads had a non-reference allele at the site. Within each track, the lower, horizontal bars represent alignment reads. Similar to above, grey means that the allele on the alignment read is the same as the reference allele at this site, colors other than grey indicate that the allele on the alignment is different from the reference allele, with blue corresponding to C, green to A, red to T and brown to G.

Candidate SNPs were accepted if the focal MA line (highlighted by the red rectangular box; see Figure S3A, S3B, S4A and S4B) was heterozygous at the focal nucleotide site indicated by the vcf file (highlighted by the red arrow; see Figure S3A, S3B, S4A and S4B), with one allele being reference and the other being non-reference, and if the genotype of the sites in the vicinity were homozygous (either for the reference allele, Figure S3A, S3B and S4A, or for a non-reference allele, Figure S4B). Candidate SNPs were rejected if the presence of the candidate SNP was in complete or complex association with variants in the vicinity (Figure S3C, S3D, S4C and S4D; also see Figure S1 and S2 in Keightley et al., 2015). These SNPs were labeled ‘FAIL’ and the reason for rejection was ‘ALIGNMENT_ARTEFACT’; these constituted 94% of all rejected SNPs. Candidate SNPs were also rejected if they fell in repetitive regions (5%, labeled ‘SOFT_MASKED_REGION’) or if they were homozygous alternate to heterozygous mutations (< 1%, labeled ‘HOMALT_TO_HET’).

**Supplementary Results**

All statistics are reported in the order of the consensus approach followed by the probabilistic approach, if not otherwise stated.

*Overview*

Of the 940 and 343 candidate mutations initially identified (i.e. before manual inspection) by the consensus approach and the probabilistic approach, 333 and 167 were found in Young T1 lines, 243 and 48 in Peak T2 lines, and 364 and 128 in Old T5 lines. After manual inspection, we accepted 69 and 154 mutations in Young Young T1 lines (11.5 ± 12.87 and 25.67 ± 9.07 per line), 23 and 43 mutations in Peak T2 lines (3.83 ± 1.38 and 7.17 ± 3.11 per line), and 83 and 122 mutations in Old T5 lines (11.83 ± 6.35 and 20.33 ± 9.10 per line; see Table S2 for details).

The acceptance rates were between 0.00% to 35.71% and 50.00% to 100.00% across lines, with an average of 19.50 ± 3.79% and 94.72 ± 1.76% in Young T1 lines, 8.64 ± 3.13% and 85.21 ± 8.04% in Peak T2 lines and 16.89 ± 5.25% and 88.14 ± 7.97% in Old T5 lines (see Table S2 for details). We investigated whether the acceptance rate candidate mutations differed between regimes. The acceptance rate of the candidate mutations called by did not differ between regimes (consensus approach: *X*^2^ = 0.04, df = 2, *p* = 0.98; probabilistic approach: *X*^2^ = 0.01, df = 2, p = 0.99).

There was no correlation between the number of callable sites and the number of mutations across lines (Pearson’s correlation; consensus approach: *r* = -0.25, *t* = -1.03, df = 16, *p* = 0.32; probabilistic approach: *r* = -0.38, *t* = -1.62, df = 16, *p* = 0.12).

*Manual inspection*

The IGV screenshots for all candidate SNPs and a file containing the acceptance or rejection, and the reason for rejection for each SNP, are available on GitHub: https://github.com/hweiyenchen/Nematode_ASM164373v4/.

*Parental-age-at-reproduction and mutation rates*

The per generation, base-substitution mutation rates range from 0.00 to 12.22 X10^-8^ (consensus approach; see Table S4 for details) and from 0.36 X10^-8^ to 22.45 X10^-8^ (probabilistic approach; see Table S4 for details), with an average of 4.23 (± 1.06) X10^-8^ and 9.45 (± 3.34) X10^-8^ in Young T1 lines, 1.36 (± 0.49) X10^-8^ and 2.55 (± 1.11) X10^-8^ in Peak T2 lines and 4.97 (± 2.28) X10^-8^ and 7.31 (± 3.27) X10^-8^ in Old T5 lines. Significant variation in mutation rates between regimes was observed by both the Poisson model and the binomial model (consensus approach: the Poisson model: *X*^2^ = 30.03, df = 2, *p* < 0.001; the binomial model: *X*^2^ = 30.80, df = 2, *p* < 0.001; probabilistic approach: the Poisson model: *X*^2^ = 54.87, df = 2, p < 0.001; the binomial model: *X*^2^ = 57.84, df = 2, *p* < 0.001).

*Mutation spectrum, transition and transversion*

No significant variation in mutation spectrum was observed among regimes (chi-square test; consensus approach: *X*^2^ = 7.97, *p* = 0.42; probabilistic approach: *X*^2^ = 14.01, *p* = 0.17). For all three regimes, significantly more transitions occurred than transversions (chi-square test; consensus approach: T1:65 transitions and 4 transversions, *X*^2^ = 53.93, *p* < 0.001; T2: 22 transitions and 1 transversion, *X*^2^ = 19.17, *p* < 0.001; T5: 77 transitions and 6 transversions, *X*^2^ = 60.74, *p* < 0.001; probabilistic approach: Young T1: 137 transitions and 17 transversions, *X*^2^ = 93.51, *p* < 0.001; Peak T2: 39 transitions and 4 transversions, *X*^2^ = 28.49, *p* < 0.001; Old T5: 109 transitions and 13 transversions, *X*^2^ = 75.54, *p* < 0.001; Table S3; Figure S6).

**Supplementary figures**

**Figure S1.** Schematic illustration of the experimental procedure.

**Figure S2.** Survival across generations for all MA regimes tested. For T1 and T5 regimes, two independent sets (set 1 and set 2) were generated at two different time points.

**Figure S3**. Exemplary IGV screenshots of candidate mutation identified by the consensus approach accepted (A and B) or rejected (C and D) by manual inspection. Red arrows indicate the location of the candidate mutation; red squares represent the panel for the MA line carrying the candidate mutation. Mutations were accepted if all reads in nearby region supported the same allele (A and B); mutations were rejected if reads in nearby region supported multiple alleles (C and D), and thus the mutation might be a misidentification resulted from alignment artefact of the region.

**Figure S4**. Exemplary IGV screenshots of candidate mutation identified by the probabilistic approach accepted (A and B) or rejected (C and D) by manual inspection. Red arrows indicate the location of the candidate mutation; red squares represent the panel for the MA line carrying the candidate mutation. Mutations were accepted if all reads in nearby region supported the same allele (A and B); mutations were rejected if reads in nearby region supported multiple alleles (C and D), and thus the mutation might be a misidentification resulted from alignment artefact of the region.

**Figure S5.** Number of single-nucleotide mutations accepted after manual inspection in each MA line. Young T1 (orange), short mutation accumulation time; Peak T2 (yellow), intermediate mutation accumulation time; Old T5 (blue), long mutation accumulation time.

**Figure S6.** Distribution of the two categories of single-nucleotide mutations of the mutation accumulation (MA) lines in three regimes identified by the two approaches: A) consensus, and B) probabilistic. Young T1 (orange), short mutation accumulation time; Peak T2 (yellow), intermediate mutation accumulation time; Old T5 (blue), long mutation accumulation time. N = 6 lines per regime. Bars show mean % of mutations across the six lines per regime with standard error (s.e.) bars.

**Supplementary tables**

**Table S1:** Mean coverage, number of callable sites, and percentage of callable genome for each MA line.

| **Regime** | **MA line ID** | **Mean coverage** | **Number of callable sites** | **Percentage of genome callable** |
| --- | --- | --- | --- | --- |
| Young T1 | T1-10 | 73.51 | 90566554 | 72.75% |
|  | T1-19 | 72.29 | 90566554 | 72.75% |
|  | T1-37 | 73.28 | 90566554 | 72.75% |
|  | T1-46 | 69.53 | 90566554 | 72.75% |
|  | T1-57 | 15.71 | 90566554 | 72.75% |
|  | T1-6 | 106.67 | 90566554 | 72.75% |
| Peak T2 | T2-1 | 123.05 | 93817854 | 75.36% |
|  | T2-22 | 54.30 | 93817854 | 75.36% |
|  | T2-34 | 83.40 | 93817854 | 75.36% |
|  | T2-4 | 44.94 | 93817854 | 75.36% |
|  | T2-42 | 59.39 | 93817854 | 75.36% |
|  | T2-52 | 94.47 | 93817854 | 75.36% |
| Old T5 | T5-45 | 43.99 | 92742405 | 74.50% |
|  | T5-5 | 62.33 | 92742405 | 74.50% |
|  | T5-70 | 57.29 | 92742405 | 74.50% |
|  | T5-81 | 53.93 | 92742405 | 74.50% |
|  | T5-86 | 50.15 | 92742405 | 74.50% |
|  | T5-87 | 49.81 | 92742405 | 74.50% |

**Table S2:** Number of variants before and after manual inspection and acceptance rate by each approach across MA lines.

| **Regime** | **MA line ID** | **Consensus approach** | | | **Probabilistic approach** | | | **Common** |
| --- | --- | --- | --- | --- | --- | --- | --- | --- |
|  |  | **Number of candidate mutations before manual inspection** | **Number of accepted mutations after manual inspection** | **Percentage of mutations accepted** | **Number of candidate mutations before manual inspection** | **Number of accepted mutations after manual inspection** | **Percentage of mutations accepted** | **Number of accepted mutations after manual inspection** |
| Young T1 | T1-10 | 56 | 20 | 35.71% | 68 | 61 | 89.71% | 4 |
|  | T1-19 | 46 | 6 | 13.04% | 15 | 14 | 93.33% | 2 |
|  | T1-37 | 51 | 9 | 17.65% | 24 | 22 | 91.67% | 2 |
|  | T1-46 | 74 | 18 | 24.32% | 47 | 44 | 93.62% | 3 |
|  | T1-57 | 86 | 14 | 16.28% | 6 | 6 | 100.00% | 1 |
|  | T1-6 | 20 | 2 | 10.00% | 7 | 7 | 100.00% | 0 |
| Peak T2 | T2-1 | 41 | 6 | 14.63% | 16 | 13 | 81.25% | 1 |
|  | T2-22 | 48 | 8 | 16.67% | 20 | 20 | 100.00% | 3 |
|  | T2-34 | 39 | 0 | 0.00% | 5 | 4 | 80.00% | 0 |
|  | T2-4 | 41 | 6 | 14.63% | 3 | 3 | 100.00% | 1 |
|  | T2-42 | 51 | 3 | 5.88% | 2 | 2 | 100.00% | 0 |
|  | T2-52 | 23 | 0 | 0.00% | 2 | 1 | 50.00% | 0 |
| Old T5 | T5-45 | 112 | 34 | 30.36% | 43 | 43 | 100.00% | 10 |
|  | T5-5 | 34 | 3 | 8.82% | 9 | 9 | 100.00% | 1 |
|  | T5-70 | 50 | 10 | 20.00% | 9 | 9 | 100.00% | 0 |
|  | T5-81 | 37 | 1 | 2.70% | 7 | 6 | 85.71% | 0 |
|  | T5-86 | 100 | 33 | 33.00% | 58 | 54 | 93.10% | 8 |
|  | T5-87 | 31 | 2 | 6.45% | 2 | 1 | 50.00% | 1 |
| **Total** | | **940** | **175** | **15.01%** | **343** | **319** | **89.36%** | **37** |

**Table S3:** Mutation rate by each approach for each MA line.

| **Regime** | **MA line ID** | **Consensus approach** | **Probabilistic approach** | **Common** |
| --- | --- | --- | --- | --- |
|  |  | **Mutation rate (X10**^-8^) | **Mutation rate (X10**^-8^) | **Mutation rate (X10**^-8^) |
| Young T1 | T1-10 | 7.36 | 22.45 | 1.48 |
|  | T1-19 | 2.21 | 5.15 | 0.74 |
|  | T1-37 | 3.31 | 8.10 | 0.74 |
|  | T1-46 | 6.63 | 16.19 | 1.10 |
|  | T1-57 | 5.15 | 2.21 | 0.37 |
|  | T1-6 | 0.74 | 2.58 | 0.00 |
| Peak T2 | T2-1 | 2.13 | 4.62 | 0.36 |
|  | T2-22 | 2.84 | 7.11 | 1.07 |
|  | T2-34 | 0.00 | 1.42 | 0.00 |
|  | T2-4 | 2.13 | 1.07 | 0.36 |
|  | T2-42 | 1.07 | 0.71 | 0.00 |
|  | T2-52 | 0.00 | 0.36 | 0.00 |
| Old T5 | T5-45 | 12.22 | 15.45 | 3.59 |
|  | T5-5 | 1.08 | 3.24 | 0.36 |
|  | T5-70 | 3.59 | 3.24 | 0.00 |
|  | T5-81 | 0.36 | 2.16 | 0.00 |
|  | T5-86 | 11.86 | 19.41 | 2.88 |
|  | T5-87 | 0.72 | 0.36 | 0.36 |

**Table S4:** Number of transitions and transversions by each approach for each MA line.

| **Regime** | **MA line ID** | **Consensus approach** | | **Probabilistic approach** | | **Common** | |
| --- | --- | --- | --- | --- | --- | --- | --- |
|  |  | **Transition** | **Transversion** | **Transition** | **Transversion** | **Transition** | **Transversion** |
| Young T1 | T1-10 | 19 | 1 | 55 | 6 | 4 | 0 |
|  | T1-19 | 6 | 0 | 13 | 1 | 2 | 0 |
|  | T1-37 | 9 | 0 | 20 | 2 | 2 | 0 |
|  | T1-46 | 16 | 2 | 38 | 6 | 3 | 0 |
|  | T1-57 | 13 | 1 | 5 | 1 | 1 | 0 |
|  | T1-6 | 2 | 0 | 6 | 1 | 0 | 0 |
| Peak T2 | T2-1 | 5 | 1 | 13 | 0 | 1 | 0 |
|  | T2-22 | 8 | 0 | 19 | 1 | 3 | 0 |
|  | T2-34 | 0 | 0 | 2 | 2 | 0 | 0 |
|  | T2-4 | 6 | 0 | 3 | 0 | 1 | 0 |
|  | T2-42 | 3 | 0 | 1 | 1 | 0 | 0 |
|  | T2-52 | 0 | 0 | 1 | 0 | 0 | 0 |
| Old T5 | T5-45 | 32 | 2 | 36 | 7 | 9 | 1 |
|  | T5-5 | 2 | 1 | 9 | 0 | 1 | 0 |
|  | T5-70 | 9 | 1 | 9 | 0 | 0 | 0 |
|  | T5-81 | 1 | 0 | 6 | 0 | 0 | 0 |
|  | T5-86 | 31 | 2 | 48 | 6 | 8 | 0 |
|  | T5-87 | 2 | 0 | 1 | 0 | 1 | 0 |
